# Literature review on the interdisciplinary biomarkers of multi-target and multi-time herbal medicine therapy to modulate peripheral systems in cognitive impairment

**DOI:** 10.3389/fnins.2023.1108371

**Published:** 2023-02-16

**Authors:** Sang-Min Park, Seung Hyun Lee, HuiYan Zhao, Jeongtae Kim, Jae young Jang, Yujin Choi, Soyeon Jeong, Soyeong Son, Kyungsook Jung, Jung-Hee Jang

**Affiliations:** ^1^College of Pharmacy, Chungnam National University, Daejeon, Republic of Korea; ^2^Interdisciplinary Program in Precision Public Health, Korea University, Seoul, Republic of Korea; ^3^KM Science Research Division, Korea Institute of Oriental Medicine, Daejeon, Republic of Korea; ^4^Korea Convergence Medical Science, Korea Institute of Oriental Medicine, University of Science and Technology, Daejeon, Republic of Korea; ^5^Department of Anatomy, Kosin University College of Medicine, Busan, Republic of Korea; ^6^School of Electrical, Electronics and Communication Engineering, Korea University of Technology and Education (KOREATECH), Cheonan-si, Republic of Korea; ^7^Functional Biomaterial Research Center, Korea Research Institute of Bioscience and Biotechnology, Jeongeup-si, Republic of Korea

**Keywords:** Alzheimer’s disease, interdisciplinary biomarker, peripheral system, syndrome differentiation, traditional medicine, herbal medicine

## Abstract

Alzheimer’s disease (AD) is a chronic progressive neurodegenerative disease characterized by the deposition of amyloid-beta (Aβ) peptide and neurofibrillary tangles in the brain. The approved drug for AD has certain limitations such as a short period of cognitive improvement effect; moreover, the development of drug for AD therapeutic single target for Aβ clearance in brain ended in failure. Therefore, diagnosis and treatment of AD using a multi-target strategy according to the modulation of the peripheral system, which is not only limited to the brain, is needed. Traditional herbal medicines can be beneficial for AD based on a holistic theory and personalized treatment according to the time-order progression of AD. This literature review aimed to investigate the effectiveness of herbal medicine therapy based on syndrome differentiation, a unique theory of traditional diagnosis based on the holistic system, for multi-target and multi-time treatment of mild cognitive impairment or AD stage. Possible interdisciplinary biomarkers including transcriptomic and neuroimaging studies by herbal medicine therapy for AD were investigated. In addition, the mechanism by which herbal medicines affect the central nervous system in connection with the peripheral system in an animal model of cognitive impairment was reviewed. Herbal medicine may be a promising therapy for the prevention and treatment of AD through a multi-target and multi-time strategy. This review would contribute to the development of interdisciplinary biomarkers and understanding of the mechanisms of action of herbal medicine in AD.

## 1. Introduction

Alzheimer’s disease (AD), a chronic progressive neurodegenerative disease marked by cognitive impairment and behavioral disorder, is characterized by the deposition of extracellular amyloid-beta (Aβ) peptide and intracellular neurofibrillary tangles, cerebral amyloid angiopathy, and neuronal loss (Sun et al., 2018). The approved drug to control AD symptoms includes two classes of drugs such as cholinesterase inhibitors and N-methyl D-aspartate (NMDA) receptor antagonist. However, they have certain limitations such that they only offer short-term cognitive improvement and cannot delay the progression of the disease (Tariot et al., 2004; Winblad et al., 2006). The development of drugs for AD with therapeutic targets including β-Site amyloid precursor protein (APP) cleaving enzyme 1 inhibitor (Kennedy et al., 2016) for Aβ clearance and immunotherapy (Honig et al., 2018) have been recently attempted. However, the efficacy of the currently being developed drugs for AD had unfavorable results in the clinical trial stage (Sun et al., 2018). In addition, the strategy of promoting Aβ clearance to improve AD may already be futile, because Aβ peptide deposits already accumulated in the brain before the onset of clinical symptoms in AD (Bateman et al., 2012). Recently, Aβ peptide in AD has been reported to be removed from the peripheral organ system as well as the brain (Xiang et al., 2015; Wang J. et al., 2017). Therefore, among several methods for the development of drugs for AD, it is necessary to develop multi-target strategies considering multiple peripheral organs for the prevention, early diagnosis, and treatment of AD that is not limited to Aβ clearance in the brain. Moreover, treatments that inhibit the progression, or delay and modify AD is still lacking (Sun et al., 2018).

Approaches through modulation of the peripheral organs by removal of Aβ peptide from the peripheral system, as well as the brain, for AD pathogenesis has been reviewed (Wang J. et al., 2017) and presents the possibility of a multi-target treatment for AD. Studies on physiological Aβ peptide clearance reported that Aβ peptide is removed from both the central and peripheral systems. The brain-derived Aβ peptide was removed in the peripheral tissues and organs in a mouse model (Xiang et al., 2015), and Aβ burden and cognitive deficits in the AD mouse model were improved by the infusion of monocytes derived from peripheral human umbilical cord blood (Darlington et al., 2015). Moreover, diseases of the peripheral organs including the liver, heart, lungs, and kidney has been suggested to be associated with Aβ clearance in AD (Wang J. et al., 2017). Hepatic dysfunction with reduced Aβ clearance by hepatocytes (Wang Y. R. et al., 2017), cardiac systolic dysfunction with reduced cerebral blood flow (Zetterberg et al., 2011), obstructive sleep apnea including decreased levels of Aβ_42_ and elevated levels of phosphorylated tau (Osorio et al., 2014), and renal dysfunction with reduced filtering Aβ from blood to urine (Liu Y. H. et al., 2015) could affect Aβ clearance. Considering these, the onset and progression of AD may be involved in peripheral systemic abnormalities. Similar to the peripheral systemic approach, traditional complementary and alternative medicine (CAM) is based on the theory of a holistic perspective and provides a personalized treatment according to a syndrome differentiation (SD) diagnostic method (Deng, 2005). SD involves a comprehensive analysis of clinical symptoms and signs to assess the cause, nature, and location of the disease based on body maladjustments and pathogenic factors for traditional therapy such as herbal medicine (HM) (Ko et al., 2014). The staged and multi-targeted sequential therapy for AD based on SD has been recently developed in Chinese medicine (Tian et al., 2019). The orderly pattern and treatment of SD according to the progression of AD starts with Shen (Kidney) deficiency to kidney-reinforcing treatment in aging, Pi (spleen) qi deficiency and Gan (liver) yin deficiency to spleen qi and liver yin-reinforcing treatment in mild cognitive impairment (MCI), progresses to phlegm and phlegm-resolving treatment in early AD, blood stasis and fire to blood activation and fire-purging treatment in middle-stage AD, and finally to severe toxin in advanced AD. A staged and multiply targeted sequential therapy combined with conventional therapy has effects at least in terms of cognitive benefit (Tian et al., 2019). Therefore, this multi-targeted and multi-time SD diagnosis and HM treatment strategy may have synergistic effects such as cognitive benefit through earlier treatment and peripheral systemic therapy. Traditional medicine treatment through SD diagnosis does not directly target the brain of patients with AD but induces the improvement of AD through a peripheral systemic approach.

The onset and progression of AD have an ordered temporal pattern, and neuropathological biomarkers are believed to precede clinical symptoms for over several decades (Jack et al., 2011). AD progression usually begins with mild cognitive impairment in the asymptomatic stage and eventually progresses to dementia and severe disability. Clinical symptoms and neurodegenerative biomarkers are closely related; therefore, the biomarkers of AD are important to characterize and monitor its progression and plan therapeutic interventions. The pathological biomarkers of AD include imaging methods to identify neurodegeneration, such as magnetic resonance imaging (MRI) and fluorodeoxyglucose-positron emission tomography (FDG PET) (Vemuri et al., 2009; Jagust et al., 2010). These diagnostic biomarkers are essential for diagnosing AD as they directly reflect its pathophysiological basis (Jack and Holtzman, 2013; Jack et al., 2018). In addition, transcriptomics is widely used for investigating the mechanisms of diseases and developing biomarkers. It is also a powerful tool that provides a convenient and reliable approach to simultaneously determine the simultaneous expression of all genes at various levels, including cells and tissues (Lowe et al., 2017). However, the limitations of obtaining target tissue samples from humans, particularly from healthy populations in general, make it difficult to study transcriptomes thereof. Therefore, non-invasive peripheral blood transcriptome is in the spotlight for its use in disease and risk assessment (Mohr and Liew, 2007). This approach is based on the assumption that the molecular profiling of circulating blood can reflect physiological and pathological events that occur in other body tissues. An individual’s overall condition can be assessed by identifying changes in peripheral gene signatures. This can contribute to future personalized healthcare strategies and lead to new methods of developing and monitoring blood biomarkers to determine an individual’s health status, disease progression, and the therapeutic effects of drugs. Research using peripheral blood transcriptome is also being actively conducted for the early diagnosis of AD and development of therapeutic markers (Hampel et al., 2012).

Analytical results using the transcriptomics approach, electroencephalogram, *in vivo* imaging, biochemical characterization, and behavioral assessments in 5XFAD mice animal models to develop therapeutic agents and biomarkers for AD have also been reported (Forner et al., 2021; Oblak et al., 2021). Recent preclinical studies have demonstrated that changes in peripheral immune cells, metabolic systems, and lesions in brain tissues are highly relevant in mouse cognitive impairment models (Bettcher et al., 2021). Aβ infiltration and nerve cell damage in the brain of AD animal models was confirmed to be greatly affected by abnormal systemic metabolism and peripheral immune cells (Bettcher et al., 2021). In relation to this, the traditional medicine approach, which restores the function of peripheral organs and induces disease recovery, has also shown efficacy in relieving symptoms through various mechanisms in cognitive impairment animal models (Wang et al., 2019; Yu et al., 2019). In addition, gene expression changes have been reported using blood transcriptome analysis in an AD mice model (Magri et al., 2021), which suggested the possibility of developing therapeutic strategies and biomarkers using the peripheral systemic approach.

Considering these findings, we aimed to review literature on multi-target for peripheral modulation that is not limited to the brain and multi-time for early diagnosis and treatment, including the pre-stage of brain damage HM treatment based on SD diagnosis. This will support the possibility of a therapeutic interdisciplinary biomarker using interdisciplinary technologies such as genetic analysis, brain imaging, and immune marker analysis. according to HM treatment in cognitive impairment.

## 2. Therapeutic effectiveness and interdisciplinary biomarker through herbal medicine therapy in patients with cognitive impairment

### 2.1. *Traditional herbal medicine therapy based on SD in mild cognitive impairment and early stage* AD

Herbal medicine significantly improved the scores in the Mini-Mental State Examination (MMSE), AD Assessment Scale-Cognitive Subscale (ADAS-cog), and Montreal Cognitive Assessment (MoCA) compared with conventional medicine alone in AD (Lee J. et al., 2022). HM has also been proven to have benefits compared with pharmaceuticals and other supportive cares in MCI, which is recognized as a pre-sign of dementia and has no approved pharmaceutical interventions (Dong et al., 2016). However, the included studies in the systematic reviews have high heterogeneity, and the HM prescriptions were largely varied. This may be because HM is personalized by SD based on various disease locations and grounded on the treatment theory of traditional medicine (Tian et al., 2019). SD in patients with cognitive impairment includes kidney deficiency, phlegm, stasis, and fire in AD and kidney deficiency, yin deficiency, heart deficiency, phlegm obstruction, Qi stagnation, and blood in MCI (Tian et al., 2019; Lin et al., 2022). The effectiveness of HM prescriptions according to the SD of Qi deficiency, kidney deficiency, and liver yang ascendant hyperactivity was identified in patients with MCI (Choi et al., 2020). In addition, SD was derived from the holistic concept including the peripheral system, and not limited to the brain (Tian et al., 2019). Therefore, HM based on SD may be possible for multi-target therapy to modulate the several peripheral organs by removing Aβ peptide not only from the peripheral system but also from brain in AD. Therefore, the efficacy of HM according to various SD classifications with a multi-target concept in AD should be investigated to more accurately evaluate the effect of HM.

A previous study on SD in elder patients with MCI suggested the distribution of six syndromes including three deficiencies (kidney, spleen qi, and heart blood) and three excesses (turbid phlegm, blood stasis, and liver depression) in MCI and suggested that the distribution ratio of deficiency and excess in complexity was higher (Yang et al., 2007). Another study has reported the correlation between SD and cognitive functions in 213 normal control, 159 patients with amnestic MCI, and 171 patients with AD (Miao et al., 2009). The syndrome score for kidney deficiency gradually significantly increased from the normal control to those with MCI and AD. The syndrome scores for turbid phlegm and blood stasis were significantly higher in those with MCI and AD than in normal control (Miao et al., 2009). Therefore, we suggested that the patterns of SD in MCI may have mixed multiple syndromes, and the SD distribution pattern may change with disease progression. In line with this, previous studies have reported about sequential therapy based on SD according to AD progression and time-order. SD according to time-order found kidney deficiency at the aging stage, spleen qi and liver yin deficiencies at the MCI stage, phlegm and qi stagnation in early AD, and blood stasis and fire heat in middle-stage AD; hence, various HM prescriptions by time-order were proposed (Tian et al., 2019). We have expressed this as multi-time therapy. In addition, retrospective analysis distinguished patients with mild- and moderate-stage AD and reported that HM combined with conventional therapy substantially decelerated the cognitive decline in AD with moderate severity and largely stabilized the cognitive function in AD with mild severity (Shi et al., 2017). Therefore, we assumed that personalized treatment by multi-target and multi-time therapy using HM based on SD according to the syndrome characteristics of individual patients and time-order progression of AD is possible in MCI and AD. In our review, the efficacy of HM based on SD by time-order including MCI and mild- and moderate-stage AD was also investigated. The search terms and databases including MEDLINE, China National Knowledge Infrastructure, and Oriental Medicine Advanced Searching Integrated System were determined through discussions among the authors.

The literature review included randomized controlled trials (RCTs) published until August 2021 in English, Korean, or Chinese. We searched for studies that classified SD and evaluated the efficacy of herbal medicine based on SD, presented the time-order progression of mild cognitive impairment and AD, and included HM interventions using traditional East Asian medicine. Observational studies, single case reports, literature reviews, re-published research papers, re-cited literature, non-SD studies, and animal studies were excluded. The search strategy is shown in Supplementary Table 1. The representative RCT studies that included studies that have large sample sizes or administered basic HM are shown in Table 1, while the remaining RCT studies are shown in Supplementary Table 2. The pattern of SD in MCI was identified as shown in the deficiency (kidney, heart, spleen, and qi and blood) and excess (phlegm and blood stasis) syndromes, as well as mixed deficiency and excess syndrome. The pattern in AD with mild to moderate stage was similar to MCI; however, excess syndrome, such as liver fire syndrome in AD or qi and blood deficiency syndrome in MCI, was only identified. In the case of patients with MCI who were classified into the four types of SD, HM prescriptions by each SD improved cognitive function. Compared to the efficacy of HM in patients with MCI and excess syndrome, MMSE and ADL greatly improved in patients with MCI and deficiency syndrome (Shen et al., 2021). The effectiveness of HM based on the heart qi and kidney essence deficiency syndrome according to AD severity showed that the effective rate of MMSE and ADL was superior in the moderate stage compared to that in the severe stage. In particular, the worse rate of MMSE and ADL was lower in CM than in WM in both the moderate and severe stages (Yu et al., 2020, 2021). This study is limited to only clinical research articles on HM efficacy based on SD that presented cognitive impairment progression. Therefore, further clinical trials or real-world data analysis such as electronic medical records studies are needed to clarify the efficacy of multi-target and multi-time therapy for cognitive impairment. By identifying the distribution and pattern of complex syndrome for individual patients and investigating the changes in SD according to AD progression, an SD system that reflects time-order, as well as customized therapy, can be created. Through this, the progression of dementia from MCI, which is the preliminary stage of dementia, may be prevented through precise medical care.

**TABLE 1:** Summaries of the included clinical research with representative syndrome differentiation in patients with cognitive impairment.

References	Stage of AD (multi-time) sample size (T/C), (HC) design	Syndrome differentiation (multi-targeting)		Treatment group period (weeks)	Control group period (weeks)	Outcome
		Type	Syndrome differentiation			
**MCI**						
Lin et al., 2020	MCI 88 (47/41), (–) RCT	Deficiency syndrome	Deficiency of heart and kidney	Tiaobu Xinshen recipe (12 weeks)	Donepezil hydrochloride (12 weeks)	No statistical difference in MMSE or MOCA in both groups Significant decrease in CM dementia syndrome score in the treatment group
Huang, 2019	aMCI 60(30/30), (–) RCT	Deficiency syndrome	Deficiency of spleen and kidney	Bu Shen Yi Zhi recipe (dose of 200 mL, twice a day) (3 months)	Aniracetam 0.2 g (for patients over 70-years-old, 0.1 g) three times a day (3 months)	Significant increase in MMSE and MoCA scores in both groups. Significant improvement in clinical memory scale, IADL scale and significant decrease in CM syndrome and AD7c-NTP in the treatment group.
Guo, 2009	MCI 64(32/32),(15) RCT	Excess syndrome	Turbid-phlegm blocking orifice syndrome	Huang Lian Wen Dan Tang, 200 mL (three times a day) Placebo capsule (3 months)	Aniracetam (three times a day) Placebo Tang (3 months)	Significant increase in MMSE, significant decrease in ADL, and more improved CM syndrome in the treatment compared with the control group More increased Ach, SOD, and MDA in the treatment group compared with the healthy control before treatment.
Shen et al., 2021	aMCI 36 (18/18), (–)	Deficiency syndrome	Deficiency of kidney essence	Di Huang Yi Zhi recipe (24 weeks)	Aniracetam 0.2 g three times a day (24 weeks)	More improved MMSE, MoCA, ADL, ADAS-Cog, CM syndrome in the treatment than in the control group
	aMCI 33(17/16), (–)	Deficiency syndrome	Deficiency of Qi and blood	Gui Pi Tang (24 weeks)	Aniracetam 0.2 g three times a day (24 weeks)	Significant increase in MMSE, MoCA score and significant decrease in ADL, ADAS-Cog, CM syndrome in both groups
	aMCI 30(15/15), (–)	Excess syndrome	Turbid-phlegm blocking orifice syndrome	Ban Xia Bai Zhu Tian Ma Tang (24 weeks)	Aniracetam 0.2 g three times a day (24 weeks)	More improved MoCA, ADAS-Cog, CM syndrome in the treatment than in the control group.
	aMCI 29(15/14), (–)	Excess syndrome	Stasis of the brain meridian and collateral	Xue Fu Zhuo Yu Tang (24 weeks)	Aniracetam 0.2 g three times a day (24 weeks)	Significant increase in MMSE and MoCA score and significant decrease in ADL, ADAS-Cog, and CM syndrome in both groups. No statistical difference in MMSE and ADL between the two groups.
**AD**						
Yu et al., 2020; Yu et al., 2021	AD mild to moderate 131(66/65), (–) RCT	Deficiency syndrome	Heart qi deficiency and kidney essence deficiency	Tiao Xin recipe Bu Shen recipe (48 weeks)	Donepezil 5 mg once daily (48 weeks)	Effective rate of MMSE was 58.97% and 58.97% in CM and WM, respectively. Worse rate of MMSE was 20.51% and 30.77% in CM and WM, respectively. Effective rate of ADL was 35.90% and 41.03% in CM and WM, respectively. Worse rate of ADL was 23.08% and 25.64% in CM and WM, respectively.
	AD severe 50(25/25), (–) RCT	Deficiency syndrome	Heart qi deficiency and kidney essence deficiency	Tiao Xin recipe Bu Shen recipe (48 weeks)	Donepezil 5 mg once daily (48 weeks)	Effective rate of MMSE was 33.33% and 23.08% in CM and WM, respectively. Worse rate of MMSE was 25.00% and 69.23% in CM and WM, respectively. Effective rate of ADL was 16.67% and 15.38% in CM and WM. Worse rate of ADL was 58.33% and 76.92% in CM and WM, respectively.
Li, 2015	AD mild to moderate 66(31/35), (–) RCT	Deficiency syndrome	Deficiency of spleen and kidney	Bu Pi Yi Shen recipe (6 months)	Donepezil 10 mg once daily (6 months)	More improved MMSE, ADL, ADAS-cog, T-AOC, SOD, MDA, 8-iso-pgf2α, and ox-LDL level in the treatment than in the control group.
Gu, 2019	AD mild to moderate 60(30/30),(–) RCT	Excess syndrome	Phlegm and blood stasis resistance network	Donepezil 5 mg once daily Jia Wei Dian Kuang Meng Xing Tang (2 months)	Donepezil 5 mg once daily (2 months)	Significant decrease in ADL and CM syndrome in both groups. Significant increase in MMSE in both groups.
Yang, 2020	AD mild to moderate 36(16/15 or 15), (–) RCT	Excess syndrome	Exuberance of heart and liver fire	Huang Lian Jie du Tang Donepezil once daily (12 weeks) (*n* = 16)	(1) Huang Lian Jie du Tang (*n* = 15) (2) Donepezil once daily (*n* = 15) (12 weeks)	Significant increase in MMSE, MOCA, ADL score, CM syndrome in the three groups Statistically different MMSE, MoCA, ADL, and CM syndrome between CM and WM.

AD7c-NTP, AD-associated neuronal thread protein; ADL, activity of daily life; ADAS-cog, Alzheimer’s disease assessment scale cognitive subscale; aMCI, amnestic MCI; CM, Chinese medicine; IADL, Instrumental Daily Living Activity; WM, Western medicine; SD, syndrome differentiation; SOD, superoxide dismutase; T-AOC, total antioxidant capacity; MMSE, Mini-Mental State Examination; MoCA, Montreal Cognitive Assessment; MDA, malondialdehyde; ox-LDL, oxidized low density lipoprotein.

**TABLE 2:** Transcriptomic biomarker studies from peripheral blood of multi-time patients with cognitive impairment.

References	Stage of AD (sample size)	Source	Data	Accession number (depository)	Findings
Han et al., 2013	Severe AD (17), mild to moderate AD (12), MCI (17), elderly CTL (13), young CTL (12)	Whole blood	Microarray	–	In the process of stage changing from MCI to mild AD to severe AD, the enrichment scores for ribosome and oxidative phosphorylation gradually decreased and increased, respectively
Chen et al., 2011	AD (5), MCI (4), CTL (4)	Blood leukocyte	Microarray	GSE18309 (GEO)	ABCB1 was the only gene that decreased in expression gradually from CTL, MCI, to AD.
Bottero and Potashkin, 2019	Set1: MCI (189), ADD (281), CTL (240) Set2: advanced ADD (9), age–matched CTL (10)	Whole blood	Microarray	Set1: GSE63063 (GEO) Set2: GSE97760 (GEO)	All the transcription factors regulating the MCI genes are shared with ADD. Pathway in MCI common with ADD: ribosome, oxidative phosphorylation, Parkinson’s disease, Alzheimer’s disease, Huntington’s disease, non-alcoholic fatty liver disease, cardiac muscle contraction. Pathway unique in MCI: retrograde endocannabinoid signaling, and protein export and metabolic pathways.
Leandro et al., 2018	Mild AD (CDR1) (15), severe AD (CDR3) (7), CTL (13)	PBMC	Microarray	E-MTAB-6094 (EMBL-EBI)	CDR1 vs. CDR3: Upregulated genes were associated with neurological process and inflammation. Downregulated genes were associated with ionic regulation, cytoskeleton, gas transport, cell signaling and olfactory transduction. GSEA results showed enriched pathways related to cell cycle regulation and neuronal function.
Moradi et al., 2019	Set1: AD (145), MCI (80), CTL (104) Set2: AD (139), MCI (110), CTL (135)	Whole blood	Microarray	Set1: GSE63060 (GEO) Set2: GSE63061 (GEO)	The selected top pathways to distinguish MCI from CTL is different from those of AD.
Song et al., 2022	AD (25), MCI (51), SCD (44), age-matched CTL (82)	Whole blood	RNA-sequencing	HRA000942 (NGDC)	SCD progressing to MCI had low interferon signaling activity. SCD with low expression of STAT1, a hub gene in the network of reconstructed interferon modules, had higher conversion rates to MCI.
Proitsi et al., 2014	AD (97), MCI (90), elderly CTL (99)	Whole blood	Microarray	–	MS4A6A expression level increased in AD but decreased in MCI compared to AD.
Shigemizu et al., 2020	AD (271), MCI (248), CTL (91)	Whole blood	RNA-sequencing	–	EEF2 and RPL7 expression levels confer the predictive model for AD which predicted a risk of MCI as well as AD conversion with a high probability.
Roed et al., 2013	MCI converters (34), stable MCI (32)	Whole blood	Microarray	–	Based on blood transcriptome data, MCI converters developing AD within 2 years could be predicted with an accuracy over 70% from stable MCI.
Lunnon et al., 2013	AD (104), MCI (118), CTL (104)	Whole blood	Microarray	–	48 genes can distinguish between AD and normal elderly control subjects with an accuracy of 75% in a validation cohort.

AD, Alzheimer’s disease; MCI, mild cognitive impairment; SCD, subtle cognitive decline; ADD, Alzheimer’s disease dementia; CTL, control; PBMC, peripheral blood mononuclear cell; GEO, gene expression omnibus; CDR, clinical dementia rating.

### 2.2. Identification of transcriptomic peripheral blood biomarkers and development of herbal medicine therapy for each stage of cognitive impairment

The expression profile in the peripheral blood of patients with AD reflects complex neuropathological conditions, including extensive systemic disruption, demonstrating strong blood-brain correlation (Naughton et al., 2015). Some studies suggested that the characteristics of MCI are similar with the pathogenesis leading to AD. A study analyzing published data on AD blood transcriptome revealed increased pathway activities associated with immune response, survival and death signaling, and cell recycling, as well as a decrease in energy metabolism-related pathways as unique as AD features (Han et al., 2013). Moreover, MCI groups showed patterns similar with AD-specific features. In particular, in the process of stages changing from MCI to mild AD to severe AD, the enrichment scores for ribosome and oxidative phosphorylation gradually decreased and increased, respectively. The expression level of ABCB1, a biomarker of AD, gradually decreased in the process of stage-changing from control to MCI to AD (Chen et al., 2011). A meta-analysis of blood transcriptome data in MCI and AD dementia (ADD) revealed that most of the genes and pathways that were altered in MCI were also altered in ADD (Bottero and Potashkin, 2019).

However, other studies have revealed that MCI and early stage AD showed unique characteristics compared to the later stages. Compared to severe AD, increased genes were mainly related to neurological processes and inflammation, and decreased genes were related to ionic regulation, cytoskeleton, gas transport, cell signaling and olfactory transduction in mild AD (Leandro et al., 2018). The results of gene set enrichment analysis showed that cell cycle regulation and neuronal function were significantly higher in mild AD. In another recent study, linear discriminant analysis was performed to identify the pathway characteristics of blood transcription that best separate AD and MCI from healthy controls (Moradi et al., 2019). The selected top pathways that distinguish MCI, including synaptic and mitochondrial function, differed from those of AD, and early pathological events in AD were thought to begin with the defects in these pathways. In a study that comprehensively evaluated blood transcriptional changes associated with subtle cognitive decline (SCD), a preclinical sign of AD, the progression of SCD to MCI had low interferon signaling activity (Song et al., 2022). Particularly, they found that individuals with low expression of STAT1, a hub gene in the network of reconstructed interferon modules, had higher conversion rates to MCI. The distinct level of MCI expressions different from AD may be related to protective mechanism (Proitsi et al., 2014). Susceptible variants in the MS4A6A gene were associated with the high risk of AD. The blood expression of MS4A6A was higher in AD compared to that in controls but slightly decreased in MCI, indicating that MCI may be an intermediate step where its expression is suppressed to minimize the adverse effects caused by MS4A6A (Proitsi et al., 2014).

Studies have also been conducted to classify the stages of patients and predict the possibility of exacerbations using machine learning techniques based on transcriptome. A predictive model for AD, which was developed using a combination of clinical, biological, and genetic properties (including EEF2 and RPL7 expression levels), also predicted a risk of MCI with high probability (Shigemizu et al., 2020). Analysis of blood gene expression in a cohort of patients with amnestic MCI showed that the group of patients with MCI developing AD within 2 years could be predicted with an accuracy of over 70% (Roed et al., 2013). In MCI, the gene expression classifier was superior to the imaging classifier in distinguishing the group diagnosed with AD within 2 years from the group that was not (Lunnon et al., 2013). These results indicate that blood transcriptome contains valuable information about the disease stage of AD and that it is possible to detect the prodromal phase.

Utilizing gene signatures that are characteristic of each stage of AD enables the establishment of appropriate drug treatment strategies for each stage. The transcriptome analysis of drug-induced changes in cells and tissues can holistically elucidate the multiple mechanisms of drugs. Therefore, it is an excellent tool for analyzing the mechanisms of HM with multi-component and multi-target properties (Liu Y. et al., 2015). Studies that have utilized transcriptome signatures, induced by the treatment of HM, have contributed to the better understanding of the various biological processes that affect AD and corroborate the implications of many previously described indications (Lee M. et al., 2022). Recently, novel molecular mechanisms, such as wound healing through focal adhesion kinase regulation by Bupleuri Radix and anticancer effects through the regulation of the Aurora B pathway by Paeoniae Radix, were proposed, based on their transcriptome signatures (Baek et al., 2022; Park et al., 2022). Furthermore, gene signature data may contribute to the development of new indications for HM (Lee M. et al., 2022). Pharmacological approach using transcriptome-based systems aim to identify drugs that show an inverse correlation with an altered disease gene signature by decreasing the expression of up-regulated genes and increasing the expression of down-regulated genes in disease models (Chen et al., 2020). Therefore, multi-time treatment strategies may be established by exploring HM prescriptions that reverse the stage-specific altered gene signature of AD. However, since studies on transcriptomic alteration by herbal medicine prescriptions treatment in cognitive impairment is still limited, further studies with multi-target and multi-time approach are needed.

In the current situation where transcriptome data is scarce, HMs effective in MCI can be explored through a network pharmacological approach, which analyses drug effects at the level of gene-drug interactions, rather than at the level of gene expression changes (Lee M. et al., 2022). In a recent study, target genes important in MCI were selected from previous literature and databases through data mining, and then herbs with potential applicability in MCI were selected by analyzing the target-component-herb network (Chang et al., 2022). In the reconstructed network, Danshen (*Salvia miltiorrhiza* bunge) and Yanhusuo (*Corydalis yanhusuo*) were the HMs that acted on most targets, and the core target genes were ADRB2, ADRA1B, DPP4, ACHE, and ADRA1D. On the other hand, a network pharmacological study on AD suggests different target genes and HMs (Fang et al., 2017), which support the need for multi-time therapy. However, such approaches based on information constructed from published literature has a limitation of bias. Therefore, experimental validation is required in the future.

### 2.3. Identification of neuroimaging biomarkers and development of herbal medicine therapy for cognitive impairment

As AD progresses, neuronal function and connectivity decrease, and the brain becomes dysfunctional. Several studies have shown that AD negatively affects normal functional network connectivity (Pievani et al., 2011; Zhu et al., 2013). One study suggested that the functional connectivity of default mode networks could be a potential AD biomarker (Balthazar et al., 2014). These findings indicate that brain functional connectivity plays an important role in diagnosing cognitive disorders, such as AD. Zhang et al. (2016) confirmed the increased connectivity in the right precuneus and global connectivity in the default mode network following long-term treatment with Bushen capsule (Zhang et al., 2016). These findings suggest that changes in brain connectivity are associated with better neuropsychological evaluation outcomes.

Neuroimaging studies that used herbal medicine therapy for cognitive impairment are shown in Table 3. AD biomarkers also include glucose metabolism as measured by FDG PET (Szelies et al., 1994), regional cerebral blood flow (rCBF), cortical and hippocampal atrophy as measured by structural MRI, and beta-amyloid levels (Smailovic et al., 2018). A study investigating the effects of toki-shakuyaku-san on MCI and AD using single photon emission computed tomography found that it significantly increased rCBF in the posterior cingulate and improved the orientation to position in patients with MCI and AD (Matsuoka et al., 2012). After 12 weeks of administrating Chotosan to patients with stroke and mild cognitive impairment, the latency of P300 (P3), an event-related potential component, was shortened, and its amplitude to novel sounds was increased in relation to the response time to sound, as well as significantly increased in the MMSE (Yamaguchi et al., 2004). Moreover, the administration of Chinese medicine (astragalus root, Prunella vulgaris, Pueraria root, lycii fructus, cnidium rhizome, rhubarb, alisma rhizome, peach kernel, ginseng, and oyster) significantly improved the MMSE score and P3 latency in comparison with that before treatment (Oishi et al., 1998). The improvement in P3 latency may be due to the improvement of dementia following Chinese medicine treatment, indicating that Chotosan improves the electrophysiological parameters of patients with mild cognitive impairment. Korean red ginseng, which has a nootropic effect, improved the frontal lobe function associated with increased relative alpha power in AD (Heo et al., 2016). When compound congrongyizhi capsule, a HM mainly used for memory decline, dementia, and contraindication, was used for treatment, the significance of the treatment group increased in the MMSE test. Functional MRI results showed increased negative brain activation in the treatment group during cognitive tasks in the posterior cingulate, inferior frontal gyrus, and lingual gyrus regions after 3 months (Zhang et al., 2014).

**TABLE 3:** Neuroimaging biomarker studies for herbal medicine treatment based on peripheral compartment in cognitive impairment patients.

References	Subjects	Intervention	Neuroimaging and neuropsychological measures	Findings
**MCI**				
Zhang et al., 2016	aMCI (*N* = 60): randomized to Bushen capsule (M:16, F:14, 66.00 ± 6.86 yrs) or Placebo (M:12, F:18, 63.33 ± 6.65 years)	4 × 3/day Bushen capsule for 24-months or 4× 3/day placebo tablet for 24-months	Neuroimaging: resting-state fMRI NTB: MMSE, episodic memory, working memory, language function, visuo-spatial ability, processing speed, and executive function	Neuroimaging: increased connectivity in the right precuneus and the global connectivity Cognition: improved general cognitive function, memory, language, and executive function especially the MMSE and episodic memory
Zhang et al., 2014	aMCI (*N* = 41) randomly divided into CCRC capsule (*N* = 16) or placebo (*N* = 12) or control (*N* = 13)	Congrongyizhi capsule and placebo group: three times/day and 4 grains for each time for 3 months, control group: no treatment	Neuroimaging: fMRI (n-back working memory task) NTB: MMSE and digit span test	Neuroimaging: increased brain negative activation in treatment group in PCC, inferior frontal gyrus and lingual gyrus regions Cognition: increased significance in the MMSE and digit span test Negative correlation between left PCC activation and changes values of MMSE and digit span test
Yamaguchi et al., 2004	MCI in stroke patients (*N* = 10, M:8, F:2, 71.3 ± 9.8 years)	7.5 g Choto-san/day between meals for 12 weeks	Neuroimaging: P300 ERP NTB: MMSE and verbal fluency test	Neuroimaging: P3 latency to target sounds was shortened in association with reduced reaction time Cognition: improved MMSE and verbal fluency test scores
**AD**				
Matsuoka et al., 2012	MCI/AD (*N* = 8, M:3, F:5, MCI:3, AD:5, 77.8 ± 4.9 years)	7.5 g toki-shakuyaku-san (TSS)/day for 8 weeks	Neuroimaging: SPECT (rCBF, resting-state) NTB: MMSE, NPI and PSMS	Neuroimaging: increased connectivity in the right precuneus and the global connectivity Cognition: improved general cognitive function, memory, language, and executive function especially the MMSE and episodic memory
Oishi et al., 1998	AD (*N* = 10, 65 ± 8 years)	Traditional Chinese medicine (Astragalus root 8 g, prunella vulgaris 3 g, Pueraria root 9 g, Lycii fructus 8 g, Cnidium rhizome 5 g, Rhubarb 1 g, Alisma rhizome 6 g, peach kernel 6 g, ginseng 3 g, oyster shell 8 g) for 3 months	Neuroimaging: P300, CT, and CSF NTB: MMSE	Neuroimaging: P300 latency improved significantly, blood flow in the cerebral cortex was improved after treatment, α-aminobutyric acid concentration in the CSF was decreased significantly after treatment Cognition: MMSE score improved significantly
Heo et al., 2016	AD (*N* = 14, M:3, F:11, 74.93 years)	4.5 g Korean red ginseng (KRG)/day for 12 weeks	Neuroimaging: EEG with quantitative spectral analysis NTB: K-MMSE and the frontal assessment battery (FAB)	Neuroimaging: alpha power increased significantly in the right temporal area, relative alpha power increased significantly in the right temporal, parietal, and occipital area Cognition: FAB score improved significantly whereas the K-MMSE showed no significant differences

AD, Alzheimer’s disease; aMCI, amnestic mild cognitive impairment; CT, computed tomography; CSF, cerebrospinal fluid; CCRC, compound congrongyizhi capsule; EEG, electroencephalography; ERP, event related potential; FAB, frontal assessment battery; F, female; fMRI, functional magnetic resonance imaging; K-MMSE, Korean mini-mental state examination; M, male; MCI, mild cognitive impairment; MMSE, mini-mental state examination; NTB, neuropsychological test battery; NPI, neuropsychiatric inventory; PSMS, physical self-maintenance scale; PCC, posterior cingulate; rCBF, regional cerebral blood flow; SPECT, single photon emission computed tomography.

Neuropsychological tests are not sensitive to precise changes in cognition. However, neuroimaging techniques provide an objective, accurate, and non-invasive measurement of neuronal function. Therefore, the use of complementary neuroimaging techniques to derive neurophysiological measures related to neural activity that underlie cognitive processes could be useful for investigating the effects of traditional HM. Nevertheless, further studies are needed to utilize neuroimaging technology as a potential biomarker for the diagnosis of AD and cognitive impairment.

## 3. Preclinical studies of herbal medicine based on the holistic theory in cognitive impairment animal models

Peripheral immune cells, as well as brain damage in the central nervous system (CNS), is known to play a key role in dementia. Bettcher et al. (2021) overviewed the role of the dysregulation of the peripheral immune system and peripheral-central immune crosstalk in AD animal models (Kato et al., 2012). AD animal model studies provide the possible mechanism of central-peripheral immune crosstalk, including the peripheral inflammatory markers (pro-inflammatory cytokines in blood level), peripheral innate immune cell infiltration (peripheral macrophages and neutrophils), immune mechanism (innate and adaptive), gut microbiome, amyloidosis, and peripheral cell infiltration (Kato et al., 2012). These experimental results show that inflammation or peripheral immune systems that occur in peripheral organs play an important role in the expression of cognitive impairments such as AD. Here, we investigated the studies that were effective in improving cognitive impairment by controlling metabolic abnormalities and systemic (peripheral and/or central) inflammation using oriental HM in mice metabolic disease and cognitive impairment models (Table 4). Preclinical studies of correlations between HM and peripheral compartments in cognitive impairment animal models comprise: (1) systemic anti-oxidation and anti-inflammation, (2) suppression of Aβ aggregation in the CNS, and (3) suppression of Aβ aggregation in the peripheral compartment.

**TABLE 4:** Preclinical studies of herbal medicine based on peripheral compartment in cognitive impairment animal models.

References	Animal models species inducer	Treatment method extracts dose/route/regimen	Test	Outcome	Potential mechanism
**Cognitive impairment animal model**					
Huang et al., 2020	APPswe/PS1dE9 double transgenic mice (30∼50 gram of body weight)	Oral administration of Danggui-Shaoyao-San (DSS) at a dosage of 3 mg/kg/day, 6.4 g/kg/day (high dose), 3.2 g/kg/day (middle dose), 1.6 g/kg/day (low dose) for 30 days	Morris water maze test, Y-maze spontaneous alternation test, open field test, fear conditioning test, biochemistry assay, western blot	Amelioration of cognition deficits restoration of abnormal levels of MDA, GSH, ROS and the activity of SOD conversion of TC, TG, LDL-c and HDL-c reduction of inflammatory factors and related enzymes	Regulation of lipid metabolism anti-oxidation anti-neuroinflammation
Pang et al., 2022	ICR mice, intracerebroventricular administration of 5 μL Aβ25-35 (5 nM/μL) (5-weeks-old)	Oral administration of ethyl acetate fraction from *Cirsium japonicum* var. *maackii* (ECJM) at a dosage of 50, 100 mg/kg/day and donepezil (positive control; 5 mg/kg/day)	Morris water maze test, T-maze test, novel object recognition test	Improvement of spatial memory, object recognition, learning and memory abilities	Inhibition of lipid peroxidation and nitric oxide production →Inhibiting of oxidative stress (Anti-oxidation)
Zhang et al., 2020	APPswe/PS1dE9 double transgenic mice (4-month-old)	Oral administration of Dengzhan Shengmai (DZSM) capsules (40, 100 mg/kg/day) and their active component scutellarin (20 mg/kg/day) for 2 months	Open field test, elevated plus maze test, Morris water maze test, histological analysis, ELISA assay, western blot	Improvement of cognitive deficits. Down-regulation of toxic soluble Aβ42 and Aβ40 level in brain cortex	Reduction of the levels of highly toxic, soluble Aβ oligomers (suppression of Aβ aggregation to lessen the level of toxic oligomers)
Liu et al., 2020	C57BL/6 mice, i.p. injection of scopolamine (8-month-old)	Oral administration of seed coat extracts of *P. suffruticosa* (PSCE; 150, 600 mg/kg/day) and donepezil (positive control; 3 mg/kg) for 4 weeks	Novel object recognition test, Morris water maze test, inhibitory avoidance test, biochemical analysis	Improvement of cognitive performance	Amelioration of cholinergic (AChE, ChAT, Ach) and oxidative damage (SOD, CAT, GSH) Anti-inflammation (IL-1β, IL-4, IL-6, and TNF-α)
Liu et al., 2022	C57BL/6 mice, oral administration of scopolamine (3 mg/kg/day) for 2 weeks (5 weeks-old)	Oral administration of Danggui-Shaoyao-San (DSS) (4.8 g/kg/day) for 1 week before scopolamine administration	Morris water maze test, novel object recognition test, serum chemistry, Western blot, 16s rRNA gene sequence analysis	Improvement of cognitive function amelioration of a shift of gut microbiota composition increase of the diversity and richness of the gut microbiota (Bacteroidetes to Firmicutes ratio)	Regulation of lipid metabolism and intestinal epithelium barrier function by modulating the abundance and diversity of gut microbiota, which plays a role in cholesterol metabolism
Gu et al., 2021	APP/PS1 transgenic mice (4 month-old)	Oral administration of Huanglian Jiedu decoction (HLJDD): Tg-APP/PS1 group (saline), HLJDD-low group (172 mg/kg/day), HLJDD-high group (344 mg/kg/day), berberine group (100 mg/kg/day), and donepezil (2 mg/kg/day).	Morris water maze test, immunohistochemistry, immunoassay, 16s rRNA gene sequence analysis	Suppression of gut dysbiosis, Aβ accumulation, neuroinflammation and reversed cognitive impairment	Anti-oxidation anti-inflammation inhibition of Aβ accumulation reduction of alteration of gut microbiome the combination of inflammatory factors (IL-6 and INF-γ), PCs and SCFA-producing bacteria were expected to be early diagnostic biomarkers for AD
Yeh et al., 2017	APP/PS1 transgenic mice hight-fat diet (60% fat) and low-dose injection of streptozotocin (50 mg/kg, i.p. injection)	Oral administration of Xuefu Zhuyu decoction (300 mg/kg) twice per day for 7 weeks after 4 weeks of HFD administration.	Nesting behavior, serum chemistry, ELISA, immunohistochemistry	Reduction of body weight, insulin and leptin level, HOMA-IR, hepatic TG, serum Aβ42 amelioration of oral glucose tolerant, Aβ deposition, astrocyte and microglia activation, and nesting behavior	Inhibition of AD-related pathology by reducing the peripheral metabolic stress mediated vascular hypoperfusion, neuroinflammation reduction of gliosis (astrocyte activation and microglia activation) suppression of cerebral Aβ accumulation
**Peripheral dysfunction animal model**					
Tang et al., 2019	C57BL/6 mice Induction of chronic renal failure (CRF) mice model by feeding of 0.2% adenine-containing diet (18∼22 gram of body weight)	Oral administration of You-Gui-Yin (YGY): CRF control group, CRF + YGY 1.5 g/kg group, CRF + YGY 3.0 g/kg group, and CRF + YGY 6.0 g/kg group for 2 weeks	Morris water maze, novel object recognition test, biochemical and physiological measurements, western blot, immunofluorescence	Amelioration of cognitive impairment. Recovery of hippocampal CaMKIIα, p-CaMKIIα (Thr286), CREB1, p-CREB1 (Ser133), and BDNF	Amelioration of cognitive impairment by up-regulating the CaMKIIα/CREB/BDNF pathway and EPO/EPOR pathways.

Ach, acetylcholine; AChE, acetylcholinesterase; BDNF, brain-derived neuotrophic factor; CAT, catalase; CaMKII, calmodulin-dependent protein kinase II; ChAT, choline acetyltransferase; CREB1, CAMP responsive dependent binding protein 1; ELISA, enzyme-linked immunosorbent assay; EPO, erythropoietin; EPOR, erythropoietin receptor; GSH, glutathione; HDL, high-density lipoprotein; HFD, high fat diet; HOMA-IR, homeostatic model assessment for insulin resistance; IL, interleukin; LDL, low-density lipoprotein; MDA, malondialdehyde; PCs, phosphatidylcholines; ROS, reactive oxygen species; SCFA, short chain fatty acid; SOD, superoxide dismutase; TC, total cholesterol; TG, triglyceride; TNF, tumor necrosis factor.

### 3.1. Systemic anti-oxidation and anti-inflammation

Several studies indicate that lipids and lipid metabolism play a role in inflammatory response (Lefterov et al., 2007; Shie et al., 2015). Specifically, omega-3 fatty acids, docosahexaenoic acid (DHA), and eicosapentaenoic acid (EPA) regulate inflammation by inhibiting polymorphonuclear leukocytes (PWN) and lowering vascular permeability, a process that may be impaired in AD (Whittington et al., 2017). The pathology of AD includes decreased DHA levels, which may result in increased brain inflammation leading to cognitive decline (Fonteh et al., 2014; Yassine et al., 2017). Other studies of AD pathogenesis indicated that the up-regulation of peripheral inflammatory cytokines is pivotal in the development of AD (Lefterov et al., 2007; Lyra et al., 2021). The oral administration of Danggui-Shaoyao-San (DSS) extract, which plays a positive effect in the up-regulation of DHA content in APPwse/PS1dE9 double transgenic mice, ameliorated cognitive deficits by anti-oxidation and anti-inflammation (Huang et al., 2020). Pang et al. (2022) reported that the oral administration of ethyl acetate fraction from *Cirsium japonicum* var. *maackii* (ECJM) inhibited lipid peroxidation and nitric oxide production in Aβ25-35-induced mice (Pang et al., 2022). Resveratrol oligomer, the seed coat extracts of *Paeonia suffruticosa*, treatments improved cognitive dysfunction in scopolamine-induced cognitive deficits in mice by inhibiting various biochemical markers, including cholinergic damage (acetyl choline esterase, choline acetyltransferase, and acetylcholine), oxidative (superoxide dismutase, catalase and glutathione) damage, and inflammatory (interleukins and tumor necrosis factor-α) pathways (Liu et al., 2020). Furthermore, Huanglian Jiedu decoction (HLJDD)-treated APP/PS1 transgenic mice showed an inhibition of Aβ accumulation in the CNS, which lead to the amelioration of cognitive impairment by anti-oxidation and anti-inflammation (Gu et al., 2021). These findings indicate that the systemic control of oxidative stress and inflammation is closely linked to the suppression of cognitive impairment in AD animal models.

### 3.2. Suppression of Aβ aggregation in the CNS

Impairment, abnormal production, and degradation of Aβ in the CNS lead to neuro-inflammation, oxidative stress, and cognitive deficits in both patients with AD and animal models. The oral administration of Dengzhan Shengmai (DZSM) capsules ameliorated cognitive impairment by up-regulating low toxic amyloid plaques and down-regulating highly toxic soluble oligomers, such as Aβ42 and Aβ40 in the brain cortex (Zhang et al., 2020). Xuefu Zhuyu decoction (XZD)-treated APP/PS1 transgenic mice with high-fat diet also showed amelioration of the suppressed cerebral Aβ aggregation, oral glucose tolerance and gliosis (activation of astrocyte and microglia) in metabolic stressed AD animal models (Yeh et al., 2017). In metabolic stress such as dysglycemia, obesity, and hepatic steatosis, it interferes with the liver’s removal of Aβ, which affects the exacerbation of AD (Yeh et al., 2017). Elevated serum Aβ levels in diabetic APP/PS1 mice were due to fatty liver, which may reduce Aβ degradation and/or excretion (Shie et al., 2015). Serum Aβ level was determined through the activity of the pathway regulating Aβ drain out from the CNS, in which liver appears to be an important player (Marques et al., 2009).

Huanglian Jiedu decoction-treated APP/PS1 transgenic mice reversed cognitive deficits with the suppression of Aβ accumulation (Gu et al., 2021). Thus, we postulate that the suppression of Aβ aggregation in the peripheral and CNS mitigates cognitive deficits in AD animal models.

### 3.3. Suppression of Aβ aggregation in peripheral compartment

The relationship between systemic inflammatory disease and AD incidence is being studied in various fields. Treatment using You-Gui-Yin (YGY) extract significantly mitigated cognitive deficits in mice with chronic renal failure (CRF) by up-regulating the CaMKIIα/CREB/BDNF and EPO/EPOR pathways in the hippocampus (Tang et al., 2019). Patients with renal failure without hemodialysis have up-regulated plasma Aβ concentration with lower cognitive function (Kato et al., 2012) because renal dysfunction with reduced Aβ filtration from blood to urine (Liu Y. H. et al., 2015). Up-regulated Aβ in the serum is closely linked to AD pathogenesis. Therefore, we postulated that YGY treatment may suppress AD progression in CRF mice through the down-regulation of Aβ accumulation in the CNS, especially in the hippocampus.

HM treatment for cognitive impairment is based on the theory of a holistic perspective that is not limited to the brain (Deng, 2005). In this review, preclinical evidence for the improvement of cognitive function by adjusting the peripheral compartment through HM was investigated. However, the basis for peripheral compartment control was limited compared to that for CNS control through HM treatment. In addition, most preclinical studies were limited to Aβ aggregation and image systems. Recently, several studies have demonstrated the association between the alteration of the microbiome and cognitive function in animals (Gareau, 2014). Furthermore, antimicrobial therapy, as used in the prevention and treatment of AD, induces the suppression of pro-inflammatory cytokines and Aβ aggregation. Treatment with DSS and HLJDD attenuated cognitive impairment through their actions on the microbiome-gut-brain axis, such as reduced alterations in the gut microbiome (Gu et al., 2021; Liu et al., 2022). In addition, Gu et al. (2021) indicated that the combination of inflammatory cytokines (interleukin-6 and interferon-gamma), phosphatidylcholines, and SCFA-producing bacteria were expected to be early diagnostic biomarkers for AD. Therefore, gut microbial alteration must also be analyzed to identify the mechanism of HM treatment for cognitive impairment.

## 4. Conclusion

Neuromodulation therapy and vagus nerve stimulation influence the CNS by accelerating motor refinement in the primary motor cortex *via* cholinergic signaling (Bowles et al., 2022). In other words, a mechanism that can improve disease without directly targeting the brain has been revealed. Similarly, the SD diagnosis for personalized HM treatment is based on the nature and location of the disease according to the holistic view of a disease that is not generalized at the site (Deng, 2005). In previous systematic reviews, HM was reported to have benefits for patients with MCI and AD (Dong et al., 2016; Lee J. et al., 2022). Moreover, RCT studies on HM prescription efficacy based on SD in MCI and AD progression time-order identified that HM improved cognitive function (Yu et al., 2020, 2021; Shen et al., 2021). Therefore, a study on the therapeutic effect mechanism of HM *via* the peripheral system approach for cognitive impairment is needed. Research on the therapeutic effect and mechanism of HM in cognitive impairment animal models showed ameliorated cognitive deficits by anti-oxidation and anti-inflammation (Huang et al., 2020; Gu et al., 2021; Pang et al., 2022). Additionally, treatment using YGY extract significantly mitigated cognitive deficits in a chronic renal failure mice model by up-regulating the CaMKIIα/CREBBDNF and EPO/EPOR pathways in the hippocampus (Tang et al., 2019). The kidney was called the “root of innate endowment” in TM; it was born with energy and gradually weakens in old age. Kidney dysfunction includes dizziness, forgetfulness, and tinnitus (The Society of Korean Medicine Diagnostics, 2012). Similar to this, in TM, the kidneys are highly related to dementia, a neurodegenerative disease. Clinical studies have reported that patients with chronic kidney disease are more prone to cognitive impairment (Shi et al., 2018). Therefore, preclinical findings on cognitive improvement and related mechanical research by HM in renal failure mice models are encouraging.

Neuroimaging-based and transcriptomic peripheral blood biomarkers were explored as HM markers by therapeutic response or disease stage progression for patients with cognitive impairment. These markers can be detected before clinical symptoms and provide an objective, accurate measurement. In particular, compared to the accumulated research on network pharmacology analysis based on the target information of phytochemicals contained in HMs (Chang et al., 2022), transcriptome-based research on molecular mechanism and neuroimaging research of HM were insufficient. Therefore, multi-time and multi-target treatment strategies must be established by exploring HM that reverse the stage-specific altered biomarkers of AD.

**FIGURE 1:**
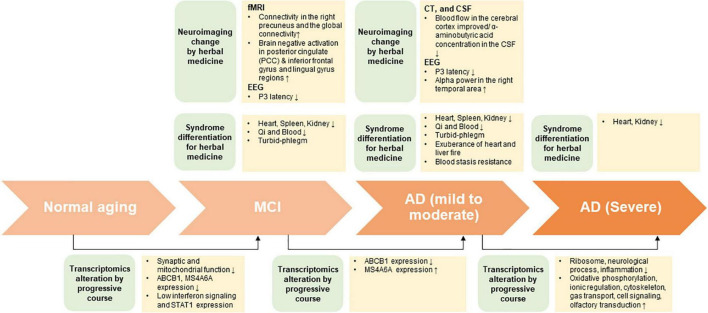
The summary on potential interdisciplinary biomarkers and syndrome differentiation diagnosis according to progressive course of cognitive impairment.

In this review, under the assumption that HM treatment is based on SD diagnosis, biomarkers of the peripheral system approach through HM treatment was presented through neuroimaging and transcriptomic changes (Figure 1). In the future, efficacy assessment, evaluation of therapeutic mechanism, and biomarker exploration must be performed for patients with cognitive impairment that were diagnosed with SD.

## Author contributions

J-HJ and KJ: conceptualization. S-MP and SL: formal analysis. JJ, YC, SJ, and SS: methodology. J-HJ: supervision. S-MP, SL, HZ, JK, KJ, and J-HJ: writing – original draft preparation. S-MP, SL, KJ, and J-HJ: writing – review and editing. All authors have read and agreed to the published version of the manuscript.
