# Supplementary material for: Literature review on the interdisciplinary biomarkers of multi-target and multi-time herbal medicine therapy to modulate peripheral systems in cognitive impairment
Source: Front Neurosci. 2023 Feb 16;17:1108371. doi: 10.3389/fnins.2023.1108371 (PMC9978226; doi:10.3389/fnins.2023.1108371)
Supplement: Supplementary file 1 [file Data_Sheet_1.docx]

Supplementary Material

# Supplementary Tables

Supplementary Table 1. Search strategy for databases

1. PubMed

| #1 | “Alzheimer disease”[MeSH Terms] |
| --- | --- |
| #2 | Cognitive Dysfunction"[Mesh] |
| #3 | “Mild cognitive impairment” |
| #4 | #1 OR #2 OR #3 |
| #5 | “pattern”[Title/Abstract] |
| #6 | “syndrome”[Title/Abstract] |
| #7 | “pattern identification”[Title/Abstract] |
| #8 | “syndrome identification”[Title/Abstract] |
| #9 | “pattern differentiation”[Title/Abstract] |
| #10 | “syndrome differentiation”[Title/Abstract] |
| #11 | “zheng”[Title/Abstract] |
| #12 | “pattern medicine”[Title/Abstract] |
| #13 | “syndrome pattern”[Title/Abstract] |
| #14 | “traditional Chinese medicine pattern”[Title/Abstract] |
| #15 | “traditional Chinese medicine syndrome”[Title/Abstract] |
| #16 | #5 OR #6 OR #7 OR #8 OR #9 OR #10 OR #11 OR #12 OR #13 OR #14 OR #15 |
| #17 | “herbal medicine”[Title/Abstract] |
| #18 | “Korea medicine”[Title/Abstract] |
| #19 | “phytotherapy”[MeSH Terms] |
| #20 | “ethnobotany”[Title/Abstract] |
| #21 | “ethnopharmacology”[Title/Abstract] |
| #22 | “traditional Chinese medicine”[Title/Abstract] |
| #23 | “Chinese medicine”[Title/Abstract] |
| #24 | “herbal formula”[Title/Abstract] |
| #25 | “herbal preparation”[Title/Abstract] |
| #26 | “decoction”[Title/Abstract] |
| #27 | #17 OR #18 OR #19 OR #20 OR #21 OR #22 OR #23 OR #24 OR #25 OR #26 |
| #28 | #4 AND #16 AND #27 |

2. CNKI (The Chinese National Knowledge Infrastructure)

| #1 | ‘阿尔茨海默病’ |
| --- | --- |
| #2 | ‘海默症’ |
| #3 | ‘认知障碍’ |
| #4 | ‘MCI’ |
| #5 | ‘Alzheimer disease’ |
| #6 | ‘AD’ |
| #7 | #1 OR #2 OR #3 OR #4 OR #5 OR #6 |
| #8 | '中药' |
| #9 | '辨证' |
| #10 | '中医辨证' |
| #11 | '脾气虚' |
| #12 | '肝阴虚' |
| #13 | 痰浊 |
| #14 | '气滞 |
| #15 | '血瘀' |
| #16 | '阳盛' |
| #17 | '火热' |
| #18 | #8 OR #9 OR #10 OR #11 OR #12 OR #13 OR #14 OR #15 OR #16 OR #17 |
| #19 | '随机' |
| #20 | '对照' |
| #21 | '安慰剂' |
| #22 | '临床疗效' |
| #23 | '疗效观察’ |
| #24 | #19 OR #20 OR #21 OR #22 OR #23 |
| #25 | '动物实验' |
| #26 | '大鼠' |
| #27 | '鼠' |
| #28 | #25 OR #26 OR #27 |
| #29 | #7 AND #18 AND #24 NOT #28 |

3.OASIS (The Oriental Medicine Advanced Searching Integrated System)

| #1 | “인지장애” OR “알츠하이머” OR “Cognitive Dysfunction” OR “Alzheimer Disease” |
| --- | --- |
| #2 | “한약” |
| #3 | #1 AND #2 |

Supplementary Table 2. Summaries of the included clinical research for the remaining syndrome differentiation not presented in Table 1.

| Study | Stage of AD (Multi-time)  Sample size (T/C), (HC)  Design | Syndrome differentiation  (Multi-targeting) | | | Treatment group  Period (weeks) | Control group  Period (weeks) | Outcome |
| --- | --- | --- | --- | --- | --- | --- | --- |
|  |  | **Type** | **Syndrome differentiation** | |  |  |  |
| MCI |  |  | |  |  |  |  |
| (2015) | MCI due to subcortical small vessel disease  54 (28/26), (33)  RCT | Deficiency syndrome | | Xin (Heart) and Shen (Kidney) deficiency | Nourishing Xin and Shen method  (12 weeks) | Donepezil  (12 weeks) | Improved MMSE, MOCA, CM dementia syndrome scales in the treatment group |
| (Qian, 2019) | MCI  63(31/32), (-)  RCT | Deficiency syndrome | | Deficiency of fluid and marrow | Tai Yuan Yin, 200 mL  (twice a day)  Donepezil, 5 mg  (12 weeks) | Donepezil, 5 mg  (once a day)  (12 weeks) | Significant increase in MMSE or MoCA score in both groups.  Significant decrease in CM syndrome in both groups. |
| (Yang, 2021) | MCI  60(34/25), (-)  RCT | Deficiency syndrome | | Deficiency of kidney essence | Hai Ma Yi Zhi powder,  (twice a day)  Donepezil, 5 mg  (once a day)  (8 weeks) | Donepezil, 5 mg  (once a day)  (8 weeks) | Significant increase in MMSE, MoCA score in both groups.  No statistical difference in the Barthel Index of both groups.  Significant decrease in CM syndrome in both groups. |
| (Chen, 2009) | MCI, mild  60(30/30),(-)  RCT | Deficiency syndrome | | Deficiency of spleen and kidney | Jia Wei Shu Yu Wan  (2 months) | Piracetam three times a day  (2 months) | No statistical difference in MMSE in both treatment group.  Significantly improved CM syndromes and ADL in both groups. |
| (Guo et al., 2010) | MCI  64 (32/32) , (-)  RCT | Excess syndrome | | Turbid-phlegm blocking orifice syndrome | modified Huanglian Wendan Decoction (dose of 200 mL, twice a day)  (12 weeks) | Aniracetam 0.2 g (for patients over 70-years-old, 0.1 g) three times a day  (12 weeks) | Increase in MMSE score in both groups  Improved CM syndrome in the treatment group  Elevated Ach serum level in treatment group |
| (Dai, 2009) | MCI  74(37/37), (-)  RCT | Excess syndrome | | Turbid-phlegm blocking orifice syndrome | Hua Tan Huo Xue recipe, 200 mL,  (twice a day)  (8 weeks) | Estazolam Tablets  (8 weeks) | Significant increase in MMSE in the treatment group  More improved PSQI in the treatment than in the control group (2, 4 weeks) |
| (Li et al., 2021a) | aMCI  60(30/30), (-)  RCT | Excess syndrome | | Turbid-phlegm blocking orifice syndrome | Modified Yuanzhi Powder  (dose of 150 mL three times a day)  (8 weeks) | Aniracetam 0.2 g three times a day  (8 weeks) | Significant increase in MMSE and MoCA scores in both groups.  Significant decrease in CM syndrome, (including heaviness of the head, loss of appetite and abdominal distention, excessive phlegm, and salivation) in both groups.  More improved MMSE, MoCA, CM syndrome, (including heaviness of the head, loss of appetite and abdominal distention, excessive phlegm, and salivation) in the treatment than in the control group |
| (Liu, 2021) | MCI  66(32/30),(-)  RCT | Mixed syndrome | | Kidney deficiency and blood stasis | Yi Shen Huo Xue tang  (twice a day)  (12 weeks) | Citicoline Sodium Capsules, 0.2 g/1 time, (three times a day)  (12 weeks) | Significant increase in MMSE and MoCA score in both groups.  Significant decrease in ADL in the treatment groups.  Significant decrease in CM syndrome in both groups.  More improved MMSE, MoCA, ADL, and CM syndrome in the treatment than in the control group. |
| (XiangDong et al., 2009) | MCI  62(32/30),(-)  RCT | Mixed syndrome | | Turbid-phlegm blocking orifice syndrome, Deficiency of spleen and kidney | Liqiao Yizhi Decoction, 400 mL,  (twice a day)  60 days | Piracetam, 400 mg  60 days | Significant increase in MMSE and MQ score in both groups.  Significant decrease in ADL in both groups.  More improved MMSE, MQ, ADL in the treatment than in the control group. |
| (Zong, 2011) | MCI, mild  166(82/82), (-)  RCT | Mixed syndrome | | Deficiency of spleen and kidney,  and phlegm and blood stasis resistance network | Shen Wu Capsule  (180 days) | Aniracetam three times a day  (180 days) | Significantly improved memory scores and CM syndromes in both groups |
| (Qiu, 2019) | aMCI  77(35/34), (-)  RCT | Mixed syndrome | | Kidney deficiency and phlegm stasis | Yi Zhi An Shen recipe  (three times a day)  (16 weeks) | Placebo  (three times a day)  (16 weeks) | Significant decrease in ADAS-cog score in the treatment group  Significant increase in MMSE and MoCA scores in the treatment group  No statistical difference in CDR-SB in both groups.  More improved PSQI, CM pattern in treatment group than control group. |
| (Gu et al., 2015) | aMCI  100(50/50), (-)  RCT | Mixed syndrome | | Kidney essence deficiency  and phlegm and blood stasis resistance network | Di Huang Yi Zhi Formula  (twice a day)  (12 weeks) | Aniracetam three times a day  (three times a day)  (12 weeks) | Significant increase in MMSE and MoCA scores in both groups.  Significant decrease in ADAS-Cog, CM syndrome in both groups.  More improved MMSE, CM syndrome (including memory loss, soreness and weakness of waist and knees, tinnitus, dream more) in the treatment group.  No statistical difference in blood routine, urine, stool routine, liver function, renal function, and cardiogram in both groups |
| (Shi, 2015) | aMCI  120(60/60), (-)  RCT | Mixed syndrome | | Marrow deficiency, Qi deficiency and blood stasis, and phlegm and blood stasis resistance network | Bu Shen Yi Jing recipe  (twice a day)  (6 months) | Donepezil, 5 mg  (once a day)  (6 months) | Significant increase in MMSE score in the treatment groups.  Significant decrease in ADAS-Cog, CM syndrome in both groups. |
| AD |  |  | |  |  |  |  |
| (Yu et al., 2012a, Yu et al., 2012b) | AD Mild to moderate  131(64/62), (-)  RCT | Deficiency syndrome | | Heart qi deficiency and Kidney essence deficiency | Tiao Xin recipe  Bu Shen recipe  (48 weeks) | Donepezil 5 mg once daily  (48 weeks) | No statistical difference in ADL scores in both groups.  Significant decrease in POD score in the treatment group. |
|  | AD severe  50(24/23), (-)  RCT | Deficiency syndrome | | Heart qi deficiency and Kidney essence deficiency | Tiao Xin recipe  Bu Shen recipe  (48 weeks) | Donepezil 5 mg once daily  (48 weeks) | Effective rate of MMSE was 70.91% and 55.77% in CM and WM, respectively.  Got worse rate of MMSE was 20.00% and 34.62% in CM and WM, respectively. |
| (Li, 2017) | AD Mild to moderate  68(31/32), (-)  RCT | Deficiency syndrome | | Deficiency of kidney and marrow | Jia Wei Shu Yu wan  (12 weeks) | Donepezil 5 mg once daily  (12 weeks) | Significant decrease in ADAS-Cog, ADL, SDSD score in the treatment group.  Significant increase in MMSE in the treatment group. |
| (Fu et al., 2012) | AD Mild  30(15/15),(-)  RCT | Deficiency syndrome | | Kidney deficiency | Yi Shen Hua Zuo recipe  (24 weeks) | Donepezil 5 mg once daily  (24 weeks) | Significant decrease in ADL in both groups.  Significant increase in MMSE in both groups. |
| (Han et al., 2016a, Han et al., 2016b) | AD Mild  40(20/20),(-)  RCT | Deficiency syndrome | | Deficiency of kidney and marrow | Bu Shen Yi Sui recipe  Placebo (Donepezil)  (24 weeks) | Donepezil 5 mg once daily  Placebo (recipe)  (24 weeks) | No statistical difference in dopamine and adrenalin in the treatment group.  Significant decrease in ADAS-Cog in the treatment group. |
| (Li et al., 2021b) | AD Mild to moderate  73(38/35), (-)  RCT | Mixed syndrome | | Kidney essence deficiency  and phlegm and blood stasis resistance network | Nao Ling powder  (4 weeks) | Donepezil 5 mg once daily  (4 weeks) | Significant increase in MMSE and HDS in the treatment group.  Significant decrease in ADL, Hcy, UA, LDL-C in the treatment group. |
| (Tong, 2014) | AD  30(15/15),(-)  RCT | Mixed syndrome | | Deficiency of spleen and kidney,  phlegm stasis | Kai Xin Jian nao Ke Li  (2 months) | Donepezil once daily  (2 months) | Significant decrease in ADL, CM syndrome in both groups.  Significant increase in MMSE in both groups. |
| (Zhang et al., 2020) | AD  Mild, moderate, and severe  68(34/34), (-)  RCT | Mixed syndrome | | Kidney deficiency and blood stasis | Shen Rong Yi Zhi Tang  (12 weeks) | Donepezil twice daily  (12 weeks) | Significant decrease in ADL in the treatment groups.  Significant increase in MMSE in the treatment groups. |
| (Zhou, 2009) | AD Mild to moderate  72(48/24),(-)  RCT | Mixed syndrome | | Deficiency of spleen and kidney,  and phlegm and blood stasis resistance network | Shen Wu capsule  (3 months) | Donepezil 5 mg once daily  (3 months) | Significant improvement in ADAS-Cog and CM syndrome in the treatment group. |

AD7c-NTP, AD-associated neuronal thread protein; ADL, Activity of daily life; ADAS-cog, Alzheimer’s disease assessment scale cognitive subscale; Apo-a, apolipoprotein a; BD, Block Design; CM, Chinese medicine; CDR-SB, Clinical dementia rating sum of box; CDT, Clock Drawing Test; CDR. Clinical Dementia Rate; Cho/Cr, Choline/Creatine; DSR, Delay Story Recall; FOM, Fuld Object-Memory Evaluation; HDS, Hasegawa dementia Scale; HBV, high whole blood viscosity; Hcy, hemocyanin; HCT, hematocrit; HMGB_1_, high mobility group box B1; IADL, Instrumental Daily Living Activity, WM, Western medicine; WMS, Wechsler Memory Scale; SD, syndrome differentiation; SOD, oxide dismutase; SDSD, dementia syndrome type scale; Tau, microtubule-associated protein; T-AOC, Total antioxidant capacity; LDL-c, Low density lipoprotein cholesterol MMSE, Mini-Mental State Examination; MoCA, Montreal Cognitive Assessment; MDA, malondialdehyde; MQ, Memory Quotient; MBP, myelin basic protein; NPI, neuropsychiatric inventory; NAA/Cr, N-acetyl aspartate/Creatine; NSE, Neuron Specific enolase; ox-LDL, oxidized low density lipoprotein; PSQI, Pittsburgh Sleep Quality index; PVS, Perivascular space; QOL-AD, quality of life in Alzheimer’s disease; RVR, Rapid Verbal Retrieve; SNSB, TG, triglyceride; 8-iso-pgf2α, UA, uric acid; 8-heterogeneous prostaglandin F2α;

Reference

2015. Nourishing Xin and Shen method improved mild cognitive impairment due to subcortical small vessel disease: a clinical study. Zhongguo Zhong Xi Yi Jie He Za Zhi*,* 35**,** 41-5. doi:10. 7661 /CJIM. 2015. 01. 0041

Chen, K. 2009. Dioscorea modified pill treatment of mild cognitive impairment (spleen and kidney deficiency type) clinical study. [master degree], [master thesis], Hubei College of Traditional Chinese Medicine.

Dai, T. 2009. Clinical Study on the Treatment of Insomnia in Patients with Mild Cognitive Impairment with the Method of Removing Phlegm, Activating Blood Circulation and Clearing Orifices. [master degree], [master thesis], Hubei College of Traditional Chinese Medicine.

Fu, K., Lin, C., Zhang, Y., Guo, J., Wang, X. & Cui, Y. 2012. Clinical Study on 15 Cases of Mild Alzheimer's Disease Treated with "Yishen Huazhuo Fang". Jiangsu Traditional Chinese Medicine*,* 44**,** 28-29. doi:10.3969/j.issn.1672-397X.2012.08.016

Gu, C., Shen, T., Mei, G., An, H., Yuan, Q., Zhang, G., et al. 2015. Clinical Observation of Using Dihuang Yizhi Formula to Treat Amnestic Mild Cognitive Impairment. Journal of Sichuan of Traditional Chinese Medicine*,* 33**,** 67-73.

Guo, R. Z., Zhou, W. Q. & Luo, Z. G. 2010. Effect of modified huanglian wendan decoction in treating senile patients with mild cognitive impairment of turbid-phlegm blocking orifice syndrome. Zhongguo Zhong Xi Yi Jie He Za Zhi*,* 30**,** 33-6.

Han, S., Gu, Y., Huang, K. & Sun, G. 2016a. Clinical Study on the Treatment of Senile Dementia of Kidney Deficiency and Marrow Deficiency with the Method of Invigorating Kidney and Benefiting Marrow. Journal of Cardiovascular and Cerebrovascular Diseases of Integrated Traditional Chinese and Western Medicine*,* 14**,** 547-548. doi:10.3969/j.issn.1672-1349.2016.05.033

Han, S., Gu, Y., Huang, K. & Sun, G. 2016b. The effect of Bushenyisui decoction on senile dementia kidney deficiency syndrome ADAS-cog point. Acta Chinese Medicine*,* 31**,** 869-872. doi:10.16368/j.issn.1674-8999.2016.06.242

Li, B., Xie, P., Guo, J., Cao, Z., Ge, Y. & Wen, Y. 2021a. Clinical Study of Modified Yuanzhi Powder Combined with Aniracetam for Amnestic Mild Cognitive Impairment( Orifices Confused by Phlegm-Turbid). Liaoning Journal of Traditional Chinese Medicine*,* 48**,** 111-113. doi:10.13192/j.issn.1000-1719.2021.02.031

Li, L., Hu, X. & Wang, L. 2021b. Clinical Effect of Naoling Decoction on Mild and Moderate AD Based on Pathogenesis of Kidney Deficiency and Phlegm and Stasis Blocking Collaterals. Acta Chinese Medicine and Pharmacology*,* 49**,** 42-46. doi:10.19664/j.cnki.1002-2392.210085

Li, X. 2017. A clinical study on mild and moderate Alzheimer's Disease of Kidney deficiency and Marrow depletion syndrome treated with modified shuyu pill. [master degree], [master thesis], Hubei university of Traditional Chinese medicine.

Liu, R. 2021. Clinical study of Yishen Huoxue decoction on Mild cognitive impairment of Kidney deficiency and blood stasis. [master degree], [master thesis], Shandong university of Traditional Chinese Medicine.

Qian, D. 2019. The clinical study on treatment of mild cognitive impairment (MCI) from deficiency of fluid. [master degree], [master thesis], Shandong university of Traditional Chinese Medicine.

Qiu, S. 2019. Clinical study of efficacy of Yi-Zhi-An-Shen formula on global cognitive performance and sleep quality of individuals with kidney deficiency, phlegm and blood stasis of aMCI. [master degree], [master thesis], Chengdu university of Traditional Chinese Medicine.

Shi, G. 2015. The clinical study of Bushenyijing square on kidney essence deficiency and phlegm and blood stasis amnestic-type mild cognitive impairment. [master degree], [master thesis], Nanjing university of Traditional Chinese Medicine

Tong, X. 2014. Clinical Observation on Kaixin Jiannao Granule in Treating Alzheimer's Disease with Spleen and Kidney Deficiency, Phlegm Turbidity and Obstruction of Orifices. [master degree], [master thesis], Hunan university of Traditional Chinese Medicine.

Xiangdong, J., Chu, X. & Zhou, Z. 2009. Clinical Study on the Effect of Liqiao Yizhi Decoction in Treating32Cases of Mild Cognitive Impairment. Journal of Traditional Chinese Medicine*,* 50**,** 308-310. doi:10.13288/j.11-2166/r.2009.04.004

Yang, S. 2021. Clinical intervention study of Haima Yizhi powder combined with denepzil hydrochioride on kidney sperm deficiency of mild cognitive impairment. [master degree], [master thesis], Xinjiang medical university.

Yu, L., Lin, S., Zhou, R., Huang, P., Dong, Y. & Wang, J. 2012a. Clinical study on improving the quality of life of patients with alzheimer's disease by tcm treatment based on syndrome differentiation. Journal of Shanghai University of Traditional Chinese Medicine*,* 26**,** 41-45. doi:10.16306/j.1008-861x.2012.04.016

Yu, L., Lin, S., Zhou, R., Tang, W., Huang, P., Dong, Y., et al. 2012b. Chinese herbal medicine fot patient with mild to moderate Alzheimer's disease based on syndrome differentiation : a randomized controlled trail. J Chin Inter Med*,* 10**,** 766-776. doi:10.3736/jcim20120707

Zhang, X., Guo, H. & Yang, R. 2020. Clinical effect of Shenrong Yizhi decoction combined with donepezil hydrochloride tablets in the treatment of Alzheimer's disease with kidney deficiency and blood stasis. Clinical Medical Research and Practice*,* 5**,** 150-152. doi:10.19347/j.cnki.2096-1413.202029056

Zhou, Q. 2009. The third stage of clinical research on senile dementia of the spleen kidney two falsely & phlegm muddly blood jam-up certificate treated with SheWu capsules. [master degree], [master thesis], Hubei College of Traditional Chinese Medicine.

Zong, J. 2011. Study on drug intervention of mild cognitive impairment in the elderly. [master degree], [master thesis], Beijing university of Traditional Chinese medicine
